# Combination of Tramiprosate, Curcumin, and SP600125 Reduces the Neuropathological Phenotype in Familial Alzheimer Disease PSEN1 I416T Cholinergic-like Neurons

**DOI:** 10.3390/ijms25094925

**Published:** 2024-04-30

**Authors:** Nicolas Gomez-Sequeda, Marlene Jimenez-Del-Rio, Carlos Velez-Pardo

**Affiliations:** Neuroscience Research Group, Faculty of Medicine, Institute of Medical Research, University of Antioquia, University Research Headquarters, Calle 62#52-59, Building 1, Laboratory 411/412, Medellin 050010, Colombia; nicolas.gomezs@udea.edu.co (N.G.-S.); marlene.jimenez@udea.edu.co (M.J.-D.-R.)

**Keywords:** Alzheimer, amyloid-beta, apoptosis, cholinergic, curcumin, i416t, mutation, sp600125, tramiprosate

## Abstract

Familial Alzheimer’s disease (FAD) is a complex and multifactorial neurodegenerative disorder for which no curative therapies are yet available. Indeed, no single medication or intervention has proven fully effective thus far. Therefore, the combination of multitarget agents has been appealing as a potential therapeutic approach against FAD. Here, we investigated the potential of combining tramiprosate (TM), curcumin (CU), and the JNK inhibitor SP600125 (SP) as a treatment for FAD. The study analyzed the individual and combined effects of these two natural agents and this pharmacological inhibitor on the accumulation of intracellular amyloid beta iAβ; hyperphosphorylated protein TAU at Ser^202^/Thr^205^; mitochondrial membrane potential (ΔΨ_m_); generation of reactive oxygen species (ROS); oxidized protein DJ-1; proapoptosis proteins p-c-JUN at Ser^63^/Ser^73^, TP53, and cleaved caspase 3 (CC3); and deficiency in acetylcholine (ACh)-induced transient Ca^2+^ influx response in cholinergic-like neurons (ChLNs) bearing the mutation I416T in presenilin 1 (PSEN1 I416T). We found that single doses of TM (50 μM), CU (10 μM), or SP (1 μM) were efficient at reducing some, but not all, pathological markers in PSEN 1 I416T ChLNs, whereas a combination of TM, CU, and SP at a high (50, 10, 1 μM) concentration was efficient in diminishing the iAβ, p-TAU Ser^202^/Thr^205^, DJ-1Cys^106^-SO_3_, and CC3 markers by −50%, −75%, −86%, and −100%, respectively, in PSEN1 I417T ChLNs. Although combinations at middle (10, 2, 0.2) and low (5, 1, 0.1) concentrations significantly diminished p-TAU Ser^202^/Thr^205^, DJ-1Cys^106^-SO_3_, and CC3 by −69% and −38%, −100% and −62%, −100% and −62%, respectively, these combinations did not alter the iAβ compared to untreated mutant ChLNs. Moreover, a combination of reagents at H concentration was able to restore the dysfunctional ACh-induced Ca^2+^ influx response in PSEN 1 I416T. Our data suggest that the use of multitarget agents in combination with anti-amyloid (TM, CU), antioxidant (e.g., CU), and antiapoptotic (TM, CU, SP) actions might be beneficial for reducing iAβ-induced ChLN damage in FAD.

## 1. Introduction

Familial Alzheimer’s disease (FAD) is the most common early-onset dementia in individuals before the age of 65 years [1]. Compared to sporadic AD (SAD, onset > 65 years of age), FAD is clinically more insidious, including greater genetic predisposition (familial mutations), greater aggressiveness, more frequent diagnosis delays, less memory impairment and greater involvement of other cognitive domains upon presentation, and greater psychosocial difficulties. Furthermore, FAD neuroimaging shows increased TAU burden, a higher frequency of hippocampal sparing and posterior neocortical atrophy, and more connectome alterations impacting frontoparietal networks than the default mode network [1,2]. These features are most probably due to genetic mutations in at least three highly penetrant genes, such as amyloid precursor protein (APP), presenilin (PSEN) 1 and 2 (https://www.alzforum.org/mutations; accessed on 4 March 2024), and summed polygenic risk variants [3]. Despite these features, FAD is neuropathologically non-distinguishable from SAD. Both FAD and SAD are characterized by the presence of at least three hallmark markers: amyloid plaques formed by the extracellular nonvascular accumulation of Aβ_42_ (eAβ_42_) peptides, neurofibrillary tangles (NFTs) formed by the intracellular accumulation of abnormal phosphorylated protein TAU [4,5], and structural degeneration of the nucleus basalis of Meynert [6]. These observations inspired the amyloid cascade theory [7,8], wherein eAβ [8,9] plays a central role in the etiology of SAD. Outstandingly, several in vivo and in vitro studies have provided evidence for Aβ-dependent TAU toxicity [10]. Therefore, Aβ is upstream of TAU in FAD pathogenesis. Based on these observations, several drugs that work by reducing amyloid plaques and/or Aβ loads are currently in clinical trials [11,12] or have already been approved by the FDA (e.g., aducanumab [13], lecanemab [14]). Although approval of these agents has advanced our knowledge of AD therapeutics with disease-modifying properties targeting amyloid [15,16]**,** further investigation is needed regarding the effectiveness, safety, and regulatory issues of monoclonal antibodies [17,18,19,20]. Interestingly, intracellular accumulation of Aβ (iAβ) as a primordial cause of neuronal deterioration has gained scientific momentum [21,22,23,24]. Therefore, other therapeutic approaches are needed (e.g., [25]). Given that FAD is molecularly a multifactorial disorder [26], it is reasonable to think that combined therapies downstream of Aβ, involving not only TAU but other molecular targets (e.g., BACE1, mitochondria, tyrosine kinases), might be more suitable to combat FAD than therapies against one target only [27,28]. In line with this thought, combinations of two symptomatic treatments, combinations of symptomatic treatments with disease-modifying therapies, or combinations of a broader range of potential disease-modifying therapies have been suggested for the treatment of AD [29]. However, a combination of potential disease-modifying therapies that target iAβ or iAβ-associated pathological pathways such as oxidative damage [30] and cell death signaling [31] might be more effective in combating FAD [28]. However, the lack of a reliable in vivo or in vitro model of FAD has hampered the development of such therapies.

Recently, our laboratory has been able to replicate neuropathological features of FAD in cholinergic-like neurons (ChLNs) caused by mutations in the catalytic unit of γ-secretase PSEN1 at codon E280A [32] and at codon I416T [33], 2 of the 11 most common pathogenic variants present in Colombia [34]. Given that most of the variants in the PSEN1 gene work as autosomal dominant negative mutations [35], it is not surprising that PSEN1 E280A and I416T shared clinical, cellular, and molecular similarities [32,33,36,37]. Indeed, both ChLN variants but not wild-type (WT) PSEN 1 ChLNs showed accumulation of iAβ, phosphorylation of TAU at pathogenic residues Ser^202^/Thr^205^, oxidative stress (OS) evidenced by the oxidation of sensor protein DJ-1 (i.e., DJ-1Cys^106^-SO_3_), and phosphorylation of transcription factor c-JUN at pathogenic residues Ser^63^/Ser^73^, as well as loss of mitochondrial membrane potential (ΔΨ_m_), overexpression of cell death signaling protein TP53, activation of caspase 3, i.e., cleaved caspase 3 (CC3), and irresponsiveness to acetylcholine (ACh)-induced Ca^2+^ ion influx [32,33]. Therefore, efficient disease-modifying approaches working on PSEN1 I416T ChLNs might also be efficient for other PSEN 1 mutations as well. 

**Figure 1:**
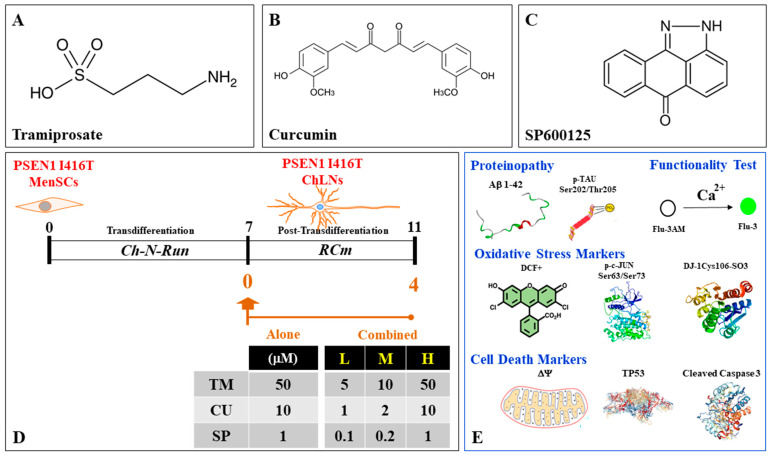
Chemical structure and molecular formula of tramiprosate (TM), curcumin (CU), and SP600125 (SP) and schematic diagram of experimental procedure. (**A**) Tramiprosate (TM), or homotaurine; 3-aminopropane-1-sulfonic acid, C3H9NO3S; (**B**) curcumin (CU), C21H20O6; (1E,6E)-1,7-bis(4-hydroxy-3-methoxyphenyl)hepta-1,6-diene-3,5-dione); (**C**) SP600125 (SP), C14H8N2O; 14,15-diazatetracyclo [7.6.1.02,7.013,16]hexadeca-1(15),2,4,6,9(16),10,12-heptaen-8-one; (**D**) PSEN1 I416T menstrual stromal cells (MenSCs) cultured in Cholinergic-N-Run (Ch-N-R) medium for 7 days transdifferentiated into cholinergic-like neurons (ChLNs). Reagents were added on day 0 (day 7 transdifferentiation) and left in culture in regular culture medium (RCm) for 4 additional days. (**E**) ChLNs were then evaluated for indictors of disease proteinopathy such as accumulation of intracellular Aβ, p-TAU Ser^202^/Thr^205^; oxidative stress (OS) (e.g., dichlorofluorescein-positive cells (DCF+) as indication of generation of reactive oxygen species (ROS), p-c-JUN Ser^63^/Ser^73^, DJ-1Cys^106^-SO_3_, cell death markers (e.g., loss of mitochondrial membrane potential (ΔΨm), overexpression of TP53 and cleaved caspase 3 (CC3)), and functionality test.

Several natural and synthetic products have been proposed to reduce the accumulation and toxic effects of Aβ peptides through different mechanisms, including antioxidant activity, secretase- or structure-dependent pathways, metal chelation, and anti-Aβ aggregation [38,39,40,41]. Tramiprosate (TM, PubChem Compound CID: 1646), also known as homotaurine, is a natural sulfonic acid (Figure 1A) found in high concentrations in green algae *Ulva lactuca* and *Gracilaria vermicullophyla* and in the brown algae *Undaria pinnatifida* [42]. Mechanistically, TM has anti-Aβ aggregation and binds to Aβ_42_ amino acid side chains Lys^16^, Lys^28^, and Asp^23^, which are responsible for both conformational seed formation and neuronal toxicity [43]. The anti-Aβ aggregation activity is further supported by the discovery of an endogenous metabolite of TM that blocks Aβ oligomer formation in the human brain [44]. In addition to reducing oligomeric and fibrillar Aβ, TM also diminished hippocampal atrophy, improved cholinergic transmission, and stabilized cognition in preclinical and clinical studies [45]. Interestingly, TM treatment in APOE4/4 homozygous patients with mild AD has been shown to stabilize cognitive performance, supporting its disease modification potential [46]. However, human clinical studies have not been conclusive regarding cognitive performance [47]. Curcumin (CU, PubChem Compound CID: 969516) is a natural polyphenol found in the rhizome of *Curcuma longa* and other *Curcuma* spp. [48]. CU contains two aryl rings containing orthomethoxy phenolic OH-groups symmetrically linked to a β-diketone (i.e., keto-enol active) moiety (Figure 1B; [49]). Due to its chemical structure, CU has shown anti-Aβ aggregation properties [50,51], antioxidant activity [52,53], metal-chelating capability [54], and diminished γ-secretase activity [55], and it inhibits pro-inflammatory molecules [56]. This observation suggests that both anti-amyloidogenic and neuroprotection actions are proprietary characteristics of TM and CU. SP600125 (Figure 1C, SP; PubChem Compound CID: 8515) is a potent and reversible inhibitor of JNK1-3 [57]. Since the JNK pathway is implicated in Aβ-induced TAU phosphorylation [32], inhibition of these kinases has been postulated as a potential target for the treatment of AD [58]. Given that these natural and synthetic products offer a multitarget approach, tagging different molecular targets in neuronal cells appears to be a more logical approach as compared with the single-target activity of most of the compounds so far used in experimental pharmacological treatments of AD [59]. We hypothesize that a combination of TM, CU, and SP might offer neuroprotection against Aβ-induced neurotoxicity in FAD. 

We therefore treated PSEN1 I416T ChLNs derived from menstrual stromal cells (MenSCs) with TM, CU, and SP individually or in combination to reduce the pathogenic FAD phenotype (Figure 1D,E). We found that combined reagents were more efficient in reverting the neuropathology markers iAβ, p-TAU Ser^202^/Thr^205^, DJ-1Cys^106^-SO_3_, p-c-JUN Ser^63^/Ser^73^, TP53, and CC3 in PSEN1 I416T than the treatment with reagents alone. Our data suggest that combined CU, TM, and SP could serve as promising therapeutic strategy for primary and secondary prevention treatments against FAD.

## 2. Results

### 2.1. Mutant Menstrual Stromal Cells (MenSCs) Transdifferentiate into PSEN 1 I416T Cholinergic-like Neurons (PSEN1 I416T ChLNs) Cultured in Ch-N-Run Medium and Further Conserve the Cholinergic Phenotype When Cultured in Regular Culture Medium

**Figure 2:**
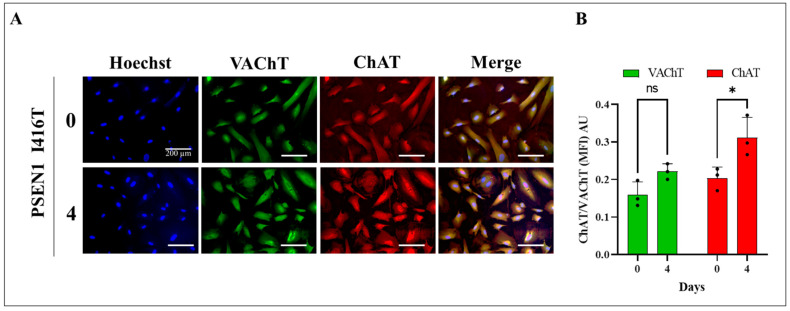
PSEN1 I416T MenSCs transdifferentiated into cholinergic-like neurons (ChLNs) express cholinergic markers choline acetyltransferase (ChAT) and vascular acetylcholine transporter (VAChT). PSEN1 I416T MenSCs were cultured with cholinergic differentiation Ch-N-Run medium for 7 days as described in the Materials and Methods section. Subsequently, they were left in regular culture medium (RCm) for 0 and 4 days. (**A**) Representative fluorescence microscopy photographs showing cholinergic ChAT (red fluorescence) and VAChT (green fluorescence) in ChLNs cultured in RCm for 0 and 4 days post-transdifferentiation. The nuclei were stained with Hoechst 33342 (blue). (**B**) Mean fluorescence intensity (MFI) quantification of images of ChAT (red fluorescence) and VAChT (green fluorescence) in ChLNs obtained by immunofluorescence analysis. The figures represent 1 out of 3 independent experiments. One-way ANOVA, post hoc test Bonferroni. Data are presented as mean ± SD, * *p* < 0.05, ns = no significance. Image magnification, 200×.

We first wanted to confirm that WT PSEN 1 and PSEN1 I416T MenSCs cultured in Ch-N-Run medium for 7 days transdifferentiated into ChLNs [33], and we tested whether the mutant ChLNs conserved the cholinergic phenotype after 4 additional days of post-transdifferentiation cultured in RCm. Effectively, fluorescence microscopy analysis shows that transdifferentiated WT (data not shown) and mutant ChLNs in Ch-N-Run expressed the typical cholinergic markers ChAT and VAChT on day 0 and day 4 post-transdifferentiation (Figure 2A,B). Considering that the mutation I416T in PSEN 1 did not affect the nature and identity of the ChLNs (from day 0 to day 4), we used those neurons obtained on day 4 post-transdifferentiation for further pharmacological experiments.

### 2.2. Tramiprosate (TM), SP600125 (SP), and Curcumin (CU) Protect PSEN 1 I416T ChLNs and Bind to Amyloid-Beta (Aβ)

**Figure 3:**
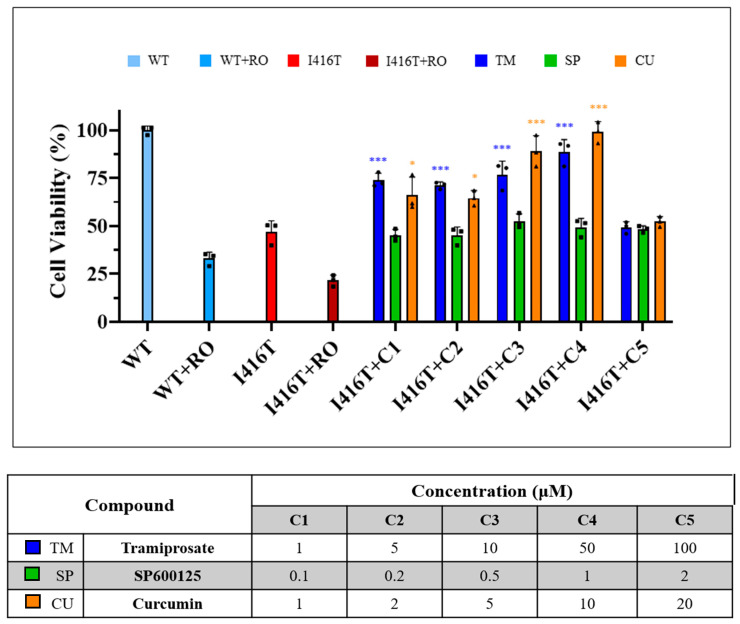
Viability of PSEN 1 I416T cholinergic-like neuron (ChLN) cells exposed to tramiprosate (TM), SP600125 (SP), and curcumin (CU), evaluated by the MTT assay. Samples were reported in comparison to the negative control (untreated wild-type cells), which was considered to have 100% viability. WT PSEN 1 and PSEN 1 E280A treated with rotenone (RO, 10 μM) were included as a positive control. Data were expressed as mean values ± SD (*n* = 3). Statistical analysis was performed using a two-way ANOVA. Data are presented as mean ± SD. Statistically significant difference as compared with the PSEN 1 I416T ChLNs at * *p* < 0.05 and *** *p* < 0.001. Bottom: Table showing the different concentrations tested of the three compounds.

Next, we evaluated whether TM, SP, and CU were toxic to PSEN 1 I416T ChLNs by using the MTT cell viability assay. Given that WT PSEN 1 ChLNs showed no markers of FAD phenotype as demonstrated previously [33], these neurons were used as a negative control (e.g., untreated WT PSEN 1 ChLNs) or as a positive control (e.g., treated with rotenone (RO, 10 μM), a specific metabolic inhibitor of mitochondrial complex I [60]). Effectively, RO significantly reduced the percentage of cell viability of both WT and mutant cells by −67% and −55%, respectively, reflecting a reduced mitochondrial metabolic activity (Figure 3). Upon exposure to TM (1, 5, 10, 50, 100 μM), SP (0.1, 0.2, 0.5, 1, 2 μM), or CU (1, 2, 5, 10, 25 μM), TM and CU up to 50 μM and 10 μM significantly increased the metabolic activity of PSEN 1 I416T ChLNs by, e.g., +89% and +111%, respectively, as an indicator of high cellular viability according to the MTT assay, whereas SP had no effect on cell viability (Figure 3). Therefore, TM at 50 μM [61], SP at 1 μM [32], and CU at 10 μM [62] were selected as the optimal concentrations for further experiments.

**Figure 4:**
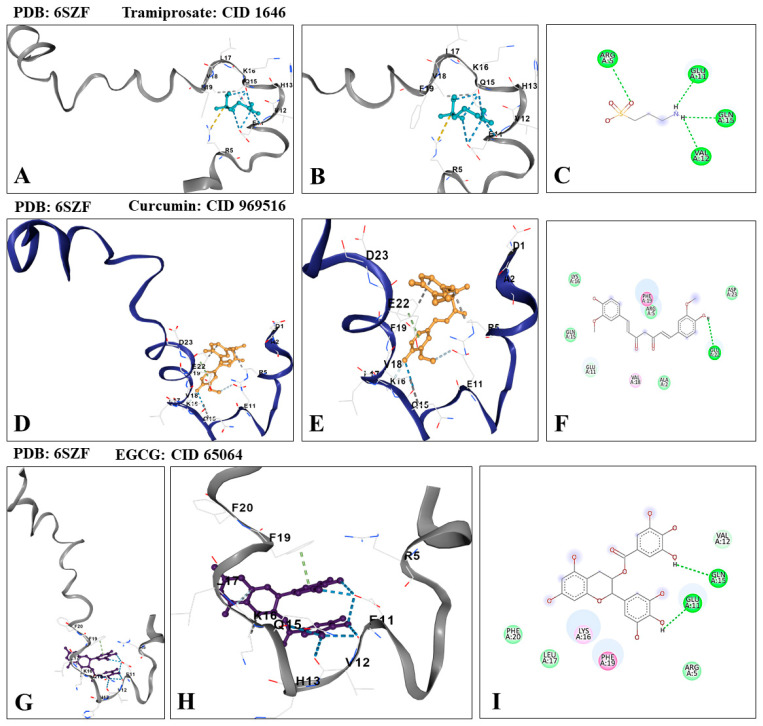
In silico molecular docking analysis of the theoretical binding of TM, CU, and epigallocatechin 3-gallate (EGCG, used as reference molecule) to monomeric Aβ. (**A**) Representative CB-Dock2 3D image showing the molecular docking of Aβ (PDB: 6SZF) with TM (CID: 1646), (**B**) close-up image of the molecular docking of Aβ with TM, (**C**) 2D diagram showing conventional hydrogen bond in Aβ-TM interaction, (**D**) representative CB-Dock2 3D image showing the molecular docking of Aβ (PDB: 6SZF) with CU (CID: 969516), (**E**) close-up image of the molecular docking of Aβ with CU, (**F**) 2D diagram showing conventional hydrogen bond in Aβ-CU interaction, (**G**) representative CB-Dock2 3D image showing the molecular docking of Aβ (PDB: 6SZF) with EGCG (CID: 65064), (**H**) close-up image of the molecular docking of Aβ with EGCG, (**I**) 2D diagram showing conventional hydrogen bond in Aβ-EGCG interaction.

Additionally, by using in silico molecular docking analysis (CB-Dock2, [63]), we calculated the theoretical binding affinity of TM and CU to monomeric Aβ_42_. For comparative purposes, the flavonoid epigallocatechin 3-gallate (EGCG; PubChem CID 65064) was included [64]. Table 1 shows the high affinity with which TM and CU interact with monomeric Aβ (PDB: 6SZF; [65]), albeit with different strengths compared to EGCG. While the binding affinity of CU (i.e., −5.6 kcal/mol Vina score) was almost similar to that of EGCG (−5.7 kcal/mol Vina score), the binding affinity of TM (−3.3 kcal/mol Vina score) was almost half of the binding affinity of EGCG (0.59) and CU (0.57). As expected, the three compounds bind to a similar binding pocket (cavity volume 283 Å^3^) and interact with similar amino acids at the N-terminus of Aβ_42_, albeit with different hydrogen bond interactions (Table 1, Figure 4A–I). 

### 2.3. Tramiprosate (TM), Curcumin (CU), and SP600125 (SP) Differentially Increase the Mitochondrial Membrane Potential (ΔΨm) and Reduce Reactive Oxygen Species (ROS) in PSEN 1 I416T ChLNs

**Figure 5:**
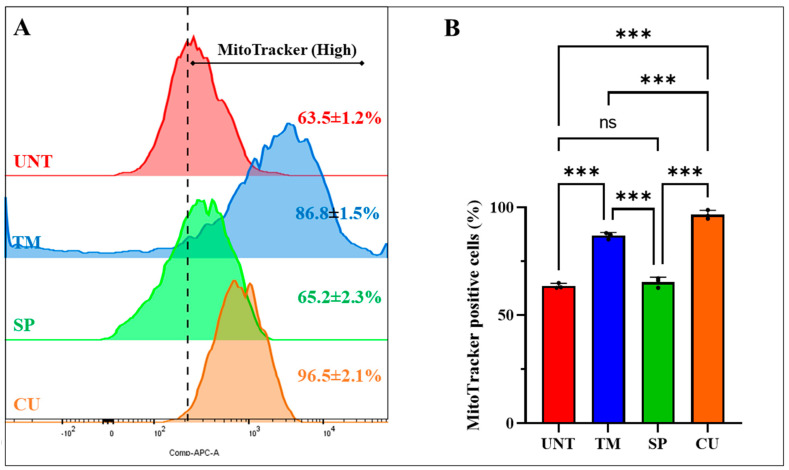

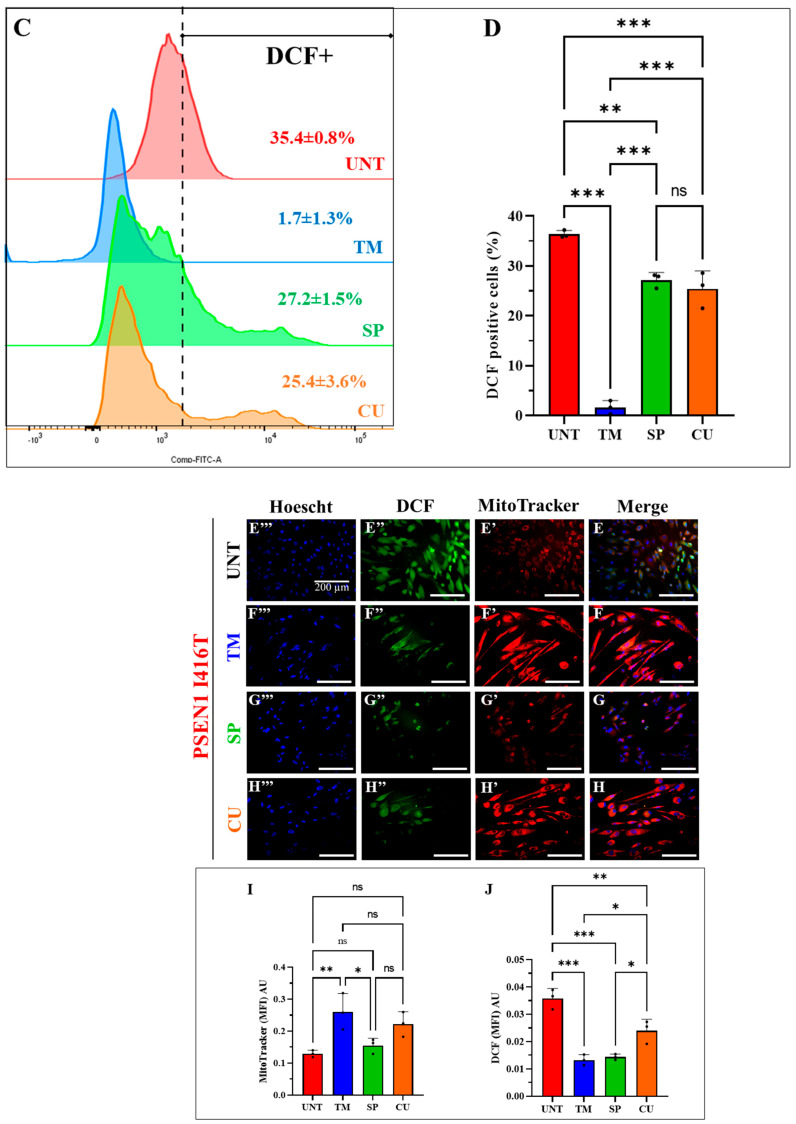
PSEN1 I416T cholinergic-like neurons (ChLNs) exposed to TM and CU showed high mitochondrial membrane potential (ΔΨm) and exhibited low levels of intracellular reactive oxygen species (ROS). PSEN1 I416T MenSCs were cultured in a cholinergic differentiation medium for 7 days as described in the Materials and Methods section. Thereafter, PSEN1 I416T ChLNs were left in regular culture medium (RCm) for 4 days exposed to TM (50 μM), SP (1 μM), and CU (10 μM). (**A**) Representative flow cytometry histogram showing the percentage decrease in ΔΨm. (**B**) Quantification of the percentage of mitoTracker-positive cells. (**C**) Representative flow cytometry histogram showing DCF-positive cells. (**D**) Quantification of DCF fluorescence intensity. (**E**–**H**) Merge fluorescence microscopy images showing triple staining with MitoTracker™ Red FM (red fluorescence, **E′**–**H′**), DCF (green fluorescence, **E″**–**H″**), and Hoechst 33342 (blue, **E**‴–**H**‴). (**I**) Quantification of ΔΨm by fluorescence intensity. (**J**) Quantification of DCF fluorescence intensity. The figures represent 1 out of 3 independent experiments. One-way ANOVA followed by Tukey’s test. Statistically significant differences: * *p* < 0.05, ** *p* < 0.01, *** *p* < 0.001, and ns = no significance. Image magnification, 200×. Figures and photomicrographs represent one out of three independent experiments (*n* = 3). The data are presented as mean ± SD of three independent experiments.

To evaluate the effect of TM, CU, and SP on the ΔΨm and generation of ROS in mutant ChLNs, cells were exposed to TM (50 μM), CU (10 μM), and SP (1 μM) only in RCm for 4 days. The experimental optimal concentration was selected according to previously published data for TM at 50 μM [61], CU at 10 μM [62], and SP at 1 μM [32]. As shown in Figure 5A, untreated mutant ChLNs show a base ΔΨm of ~64% (Figure 5A). While CU significantly increased ΔΨm by +52% and TM by +36% in PSEN 1 I416T ChLNs, SP showed a similar (low) ΔΨm (65%) to that of untreated mutant ChLNs according to flow cytometry analysis (Figure 5B). Flow cytometry analysis also shows that mutant ChLNs endogenously produced 35% DCF+ cells as evidence of ROS generation (Figure 5C, [33]), but treatment with TM dramatically reduced ROS by −94%. The treatment of cells with SP and CU modestly reduced the percentage of ROS by −22% and −29% DCF+, respectively. Similar observations were obtained by fluorescence microscopy (Figure 5E–J).

To further ascertain that the reduction in ROS was due to antioxidant activity, TM and CU were evaluated with ORAC and FRAP assays. Accordingly, CU showed a FRAP assay value of 702,184.27 ± 22,161.69 μmol Trolox equivalents (TE)/g, whereas the ORAC assay showed a value of 12,205.65 ± 456.53 μmol Trolox equivalents (TE)/g. TM showed a FRAP value of 15.35 ± 1.27 μmol TE/g and an ORAC value of 0.98 ± 0.02 μmol TE/g.

### 2.4. Tramiprosate (TM), Curcumin (CU), and SP600125 (SP) Differentially Decrease the Oxidized Protein DJ-1 and Accumulation of iAβ in PSEN 1 I416T ChLNs

**Figure 6:**
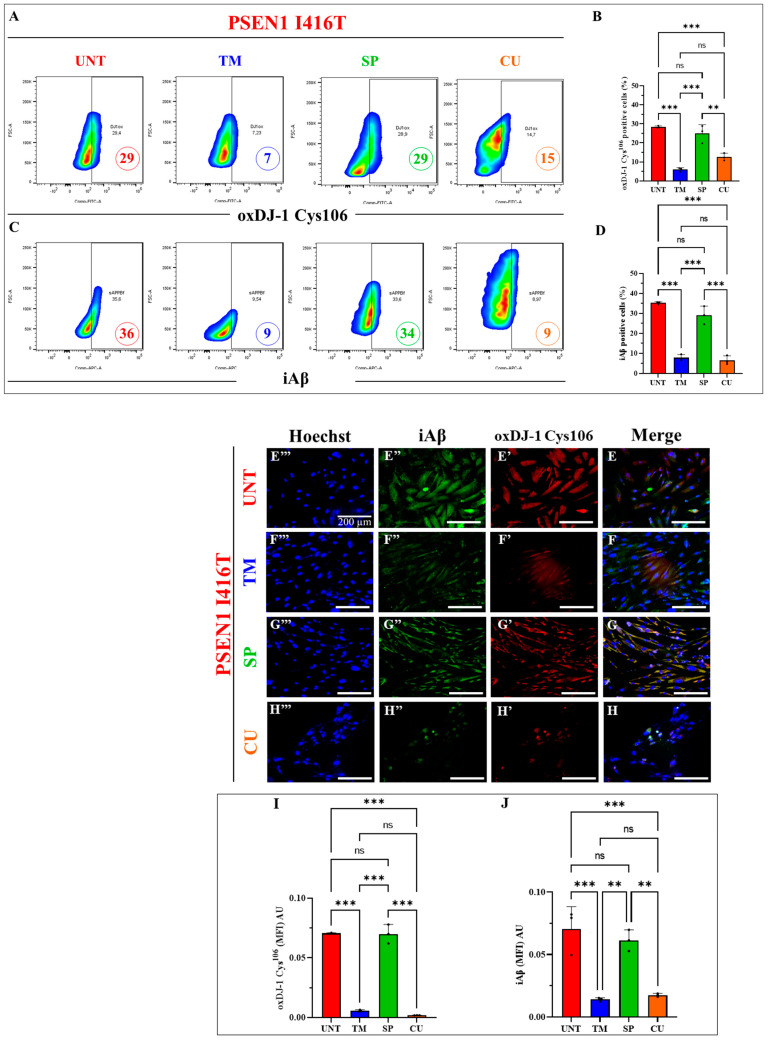
PSEN1 I416T cholinergic-like neurons (ChLNs) treated with TM and CU exhibit a reduction in intracellular Aβ and oxidized DJ-1. PSEN1 I416T MenSCs were cultured in a cholinergic differentiation medium for 7 days as described in the Materials and Methods section. Subsequently, PSEN1 I416T ChLNs were maintained in regular culture medium (RCm) for 4 days untreated or supplemented with TM (50 μM), SP (1 μM), and CU (10 μM) only. (**A**) Representative density plot depicting the percentage of oxDJ-1-Cys^106^-SO_3_. (**B**) Quantification of the percentage of oxDJ-1-Cys^106^-SO_3_-positive cells. (**C**) Representative flow cytometry density plot illustrating the percentage of iAβ. (**D**) Quantification of the percentage of iAβ-positive cells. (**E**–**H**) Merge fluorescence microscopy imagine showing double staining with antibodies against oxDJ-1-Cys^106^-SO_3_ (red, **E′**–**H′**) and iAβ (green, **E″**–**H″**). Nuclei were stained with Hoechst 33342 (blue, **E**‴–**H**‴). (**I**) Mean fluorescence intensity (MFI) quantification of oxDJ-1-Cys^106^-SO_3_. (**J**) Mean fluorescence intensity (MFI) quantification of iAβ. Figures and photomicrographs serve as illustrative examples from one of the three independent experiments (*n* = 3). The results are reported as the mean ± standard deviation of three independent experiments. Statistical analysis involved a one-way ANOVA followed by Tukey’s test. Statistical significance is indicated by ** *p* < 0.01, *** *p* < 0.001, and ns = no significance. The images were magnified at 200×.

It is well established that the ROS, i.e., H_2_O_2_, specifically oxidizes the residue Cys^106^SH (sulfhydryl group) of the stress sensor protein DJ-1 into DJ-1-Cys^106^SO_3_ (sulfonic group, [66]). Since DJ-1-Cys^106^-SO_3_ constitutes a biomarker of cellular OS [67], we wanted to confirm that mutant cholinergic neurons endogenously produce H_2_O_2_ (ROS), thereby displaying DJ-1-Cys^106^SO_3_ [33], and to test whether the reagents individually interfere with such oxidation. As shown in Figure 6, mutant ChLNs display 29% oxidized DJ-1-positive cells according to a flow cytometry assay (Figure 6A,B). TM and CU treatment reduced oxDJ-1 by −76% and −48%, respectively, whereas no significant change in oxDJ-1 was induced by the inhibitor SP (Figure 6A,B). On the other hand, PSEN1 I416T effectively accumulated iAβ by 36%. TM and CU treatment dramatically reduced the accumulation of iAβ by −75%, whereas no statistical differences were observed with SP treatment compared to untreated cells (Figure 6C,D). Similar data were obtained by fluorescence microscopy analysis (Figure 6E–J).

### 2.5. Tramiprosate (TM), Curcumin (CU), and SP600125 (SP) Differentially Decrease Phosphorylation of Protein TAU and Transcription Factor c-JUN in PSEN 1 I416T ChLNs

**Figure 7:**
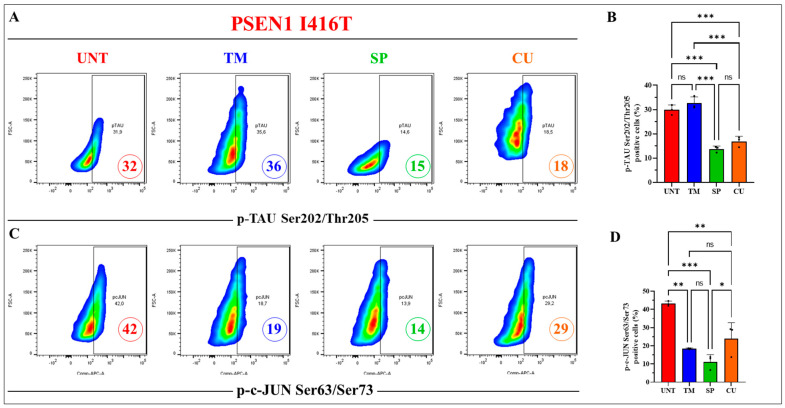

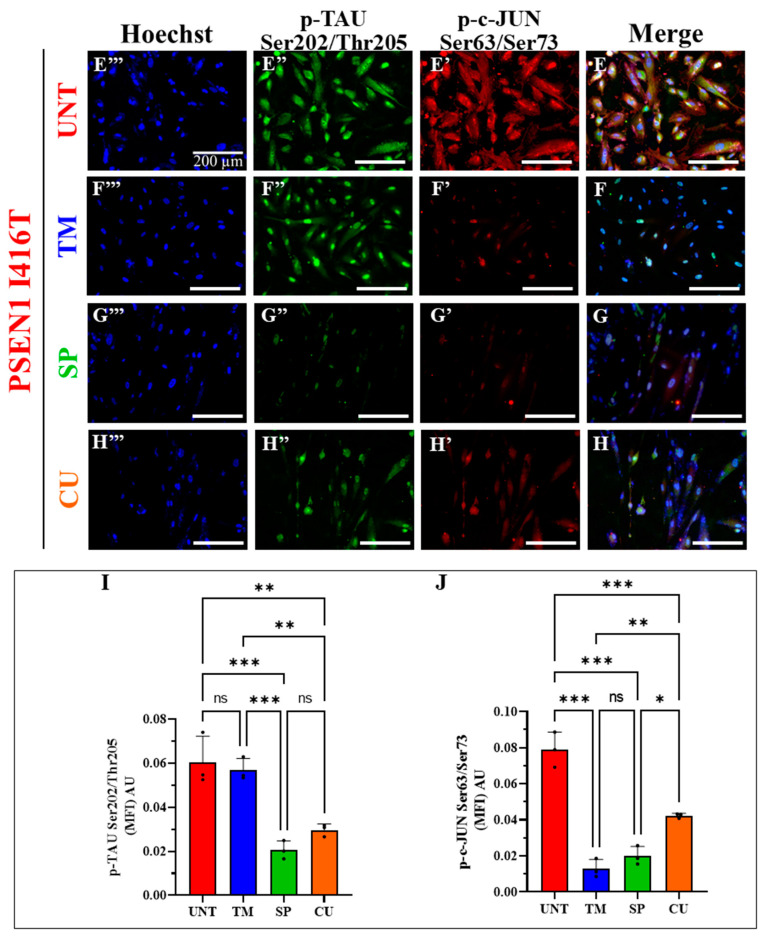
PSEN1 I416T cholinergic-like neurons (ChLNs) treated with SP and CU display reduced phosphorylation of protein TAU and c-JUN. PSEN1 I416T MenSCs were cultured for 7 days in a cholinergic differentiation medium following the protocols outlined in the Materials and Methods section. Subsequently, PSEN1 I416T ChLNs underwent a 4-day culture in standard culture medium (RCm) supplemented with TM (50 μM), SP (1 μM), and CU (10 μM). (**A**) Representative flow cytometry density plots illustrating phosphorylated TAU (p-TAUSer^202^/Thr^205^). (**B**) Quantification of the percentage of cells exhibiting p-TAUSer^202^/Thr^205^-positive staining. (**C**) Representative density plot from flow cytometry data showing phosphorylated c-JUN (p-c-JUNSer^63^/Ser^73^). (**D**) Quantification of the percentage of p-c-JUNSer^63^/Ser^73^-positive cells. (**E**–**H**) Merge fluorescence microscopy imagine showing double staining with antibodies specific for p-c-JUNSer^63^/Ser^73^ (red, **E′**–**H′**) and p-TAUSer^202^/Thr^205^ (green, **E″**–**H″**). Nuclei were stained with Hoechst 33342 (blue, **E**‴–**H**‴). (**I**) Mean fluorescence intensity (MFI) for p-TAU Ser^202^/Thr^205^. (**J**) Mean fluorescence intensity (MFI) for p-c-JUNSer^63^/Ser^73^. Figures and micrographs represent one out of three conducted experiments. The results are reported as the mean ± standard deviation of 3 independent experiments. A one-way ANOVA, followed by Tukey’s test, was conducted for statistical analysis. Statistically significant variations are indicated by * *p* < 0.05, ** *p* < 0.01, *** *p* < 0.001, and ns = no significance. The images were magnified at 200×.

Previous studies have shown that JNK inhibitor SP inhibited the phosphorylation of c-Jun [57] and phosphorylation of TAU [32]. Therefore, we wondered whether SP, TM, or CU might reduce p-TAU at Ser^202^/Thr^205^ and p-c-JUN at residue Ser^63^/Ser^73^. As expected, the specific JNK inhibitor SP reduced both p-TAU by −53% (Figure 7A,B) and p-c-JUN by −67% (Figure 7C,D), while CU reduced p-TAU by −43% and p- JUN by −31%. TM treatment had almost no effect on p-TAU compared to untreated cells but diminished p-c-JUN by −55% (Figure 7A–D). Similar information was found by immunofluorescence microscopy (Figure 7E–J).

### 2.6. Tramiprosate (TM), Curcumin (CU), and SP600125 (SP) Differentially Decrease the Apoptosis Signaling Transcription Factor TP53 and Executer Protein Caspase 3 (CASP3) in PSEN 1 I416T ChLNs

**Figure 8:**
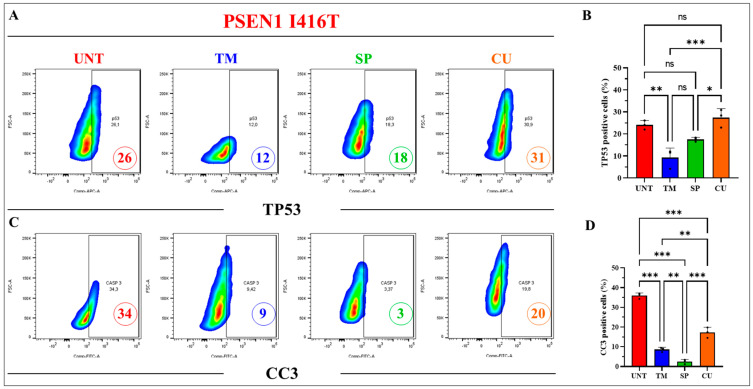

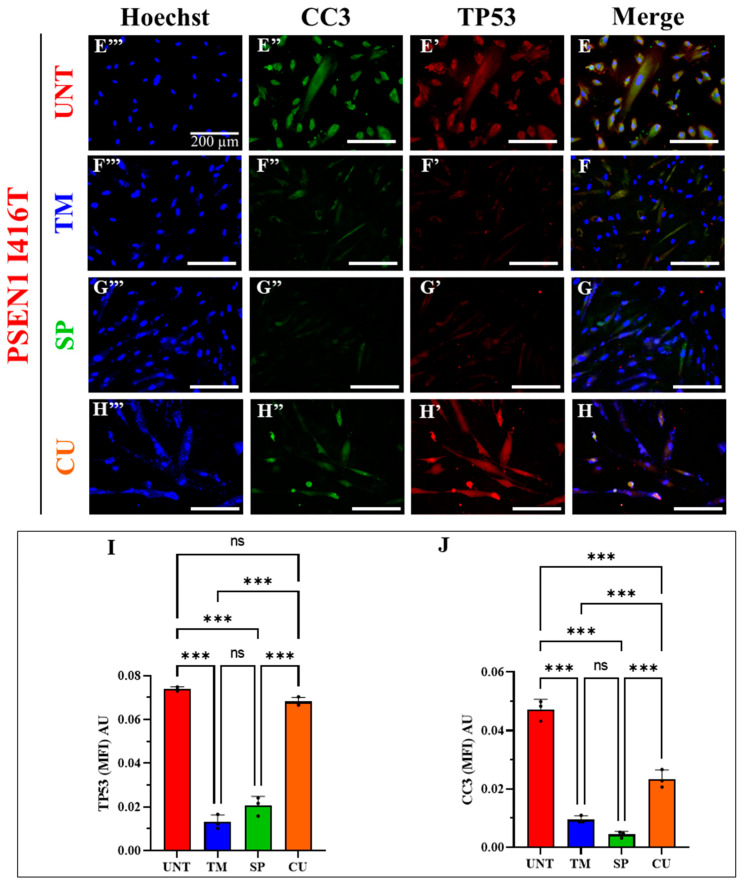
PSEN1 I416T cholinergic-like neurons (ChLNs) treated with TM, SP, and CU present a reduction in the expression of TP53 and apoptotic marker cleaved caspase 3 (CC3). PSEN1 I416T MenSCs were cultured for 7 days in a cholinergic differentiation medium following the procedures outlined in the Materials and Methods section. Subsequently, PSEN1 I416T ChLNs were cultured in regular culture medium (RCm) for 4 days and supplemented with TM (50 μM), SP (1 μM), and CU (10 μM). (**A**) Representative flow cytometry density plots depicting percentage of TP53. (**B**) Quantification of the percentage of TP53-positive cells. (**C**) Representative density plot from flow cytometry results illustrating percentage of CC3. (**D**) Quantification of the percentage of CC3-positive cells. (**E**–**H**) Merge fluorescence microscopy imagine showing double staining with antibodies against TP53 (red, **E′**–**H′**) and CC3 (green, **E″**–**H″**). Nuclei were stained with Hoechst 33342 (blue, **E**‴–**H**‴). (**I**) Mean fluorescence intensity (MFI) for TP53. (**J**) Mean fluorescence intensity (MFI) for CC3. Histograms and photomicrographs represent one of the three independent experiments. The results are reported as the mean ± standard deviation of 3 independent experiments. A one-way ANOVA followed by Tukey’s test was conducted for statistical analysis. Statistically significant are indicated by * *p* < 0.05, ** *p* < 0.01, *** *p* < 0.001, and ns = no significance. The images were magnified at 200×.

Next, we wanted to determine whether TM, CU, and SP could modulate the expression of apoptogenic protein TP53 and activation of CASP3 (i.e., cleaved caspase 3, CC3) as observed in PSEN1 I416T ChLNs [33]. Figure 8 shows that all three agents affected the expression of TP53, albeit with different strengths. While TM and SP reduced TP53 to 12% (−54%) and 18% (−31%) compared to untreated cells (26%), CU slightly increased TP53 to 31% (+19%, Figure 8A,B). Interestingly, all three agents consistently reduced CC3. TM, SP, and CU abridged CC3 by −74%, −91%, and −41% (Figure 8C,D). Similar results were obtained by immunofluorescence microscopy (Figure 8E–J). 

### 2.7. Tramiprosate (TM), Curcumin (CU), and SP600125 (SP) Differentially Increase Ca^2+^ Influx Induced by Acetylcholine (ACh) in PSEN 1 I416T ChLNs

**Figure 9:**
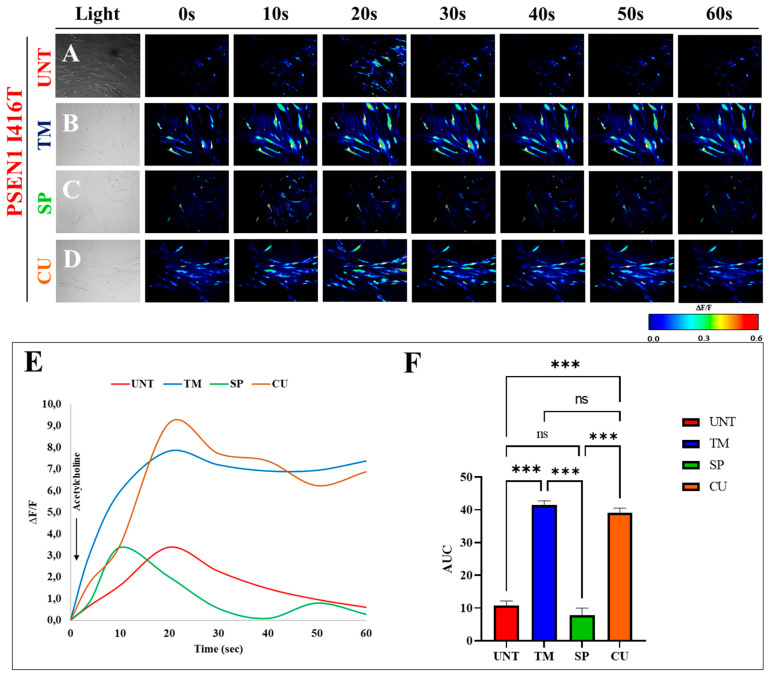
PSEN1 I416T ChLNs treated with CU or TM exhibit an increase in functional response to acetylcholine (ACh). After 7 days of transdifferentiation, PSEN1 I416T ChLNs were cultured in regular culture medium for 4 days and treated with one of the following substances: TM (50 μM), SP (1 μM), and CU (10 μM), as indicated in the figure. ACh was puffed into the culture at 0s (arrow). Then, the Ca^2+^ fluorescence of the cells was monitored at the specified times. Color contrast indicates fluorescence intensity: dark blue < light blue < green < **yellow** < red. Representative time-lapse images (0, 10, 20, 30, 40, 60 s) of Ca^2+^ fluorescence in response to ACh in PSEN1 I416T ChLNs after 4 days of (**A**) untreated conditions or treatment with (**B**) TM, (**C**) SP, (**D**) CU. (**E**) Graph showing ΔF/F and (**F**) area under the curve (AUC) of cells with and without treatment. The figures represent 1 out of 3 independent experiments. Data are expressed as the mean ± SD; *** *p* < 0.001, ns = no significance. Image magnification, 200×.

PSEN 1 I416T ChLNs have shown a lessened functional response to ACh-induced transient Ca^2+^ influx [33]. To test whether TM, SP, and CU might restore cholinergic functionality, mutant ChLNs were exposed to ACh (1 mM final concentration) alone or in pre-cultured ChLNs with the single treatment of reagents. As expected, mutant ChLNs show a dysfunctional Ca^2+^ influx response to ACh (Figure 9A) with an average fluorescence change (ΔF/F) = 3.39 ± 0.94 and a mean duration of 20 s each (*n* = 20 ChLN cells imaged, N = 3 dishes) according to cytoplasmic Ca^2+^ responses to Fluo-3-mediated imaging (Figure 9E,F). While SP was completely ineffective at reverting ACh-induced transient Ca^2+^ influx in mutant ChLNs (Figure 9C), TM (Figure 9B) and CU (Figure 9D) were capable of restoring PSEN 1 I416T ChLNs’ responsivity to ACh with an average fluorescence change (ΔF/F) = 7.82 ± 0.55 and 9.12 ± 0.83 (Figure 9E) and a mean duration of 20 s each (*n* = 20 ChLN cells imaged, N = 3 dishes) as intracellular Ca^2+^ responses (Figure 9F).

### 2.8. Combination of Tramiprosate (TM), Curcumin (CU), and SP600125 (SP) at High (H) or Middle (M) but Not Low (L) Concentration Reduces iAβ, p-TAU, oxDJ-1, and CC3 in PSEN 1 I416T ChLNs

**Figure 10:**
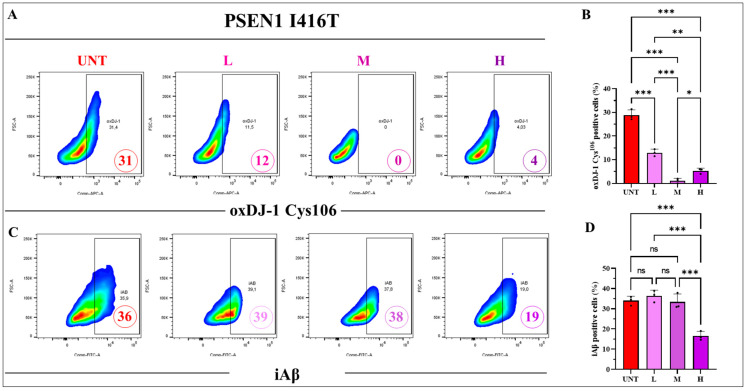
PSEN 1 I416T ChLNs treated with the combination of TM, CU, and SP at low (5, 1, 0.1), middle (10, 2, 0.2), or high (50, 10, 1) concentrations (μM) demonstrate a significant decrease in the oxidation of DJ-1 and production of intracellular Aβ (iAβ). PSEN1 I416T MenSCs were transdifferentiated and subjected to combined treatments of SP, TM, and CU for 4 days at low (L), middle (M), and high (H) concentrations, following the protocols outlined in the Materials and Methods section. (**A**) Representative flow cytometry density plot illustrating the percentage of oxDJ-1-Cys^106^-SO_3_-positive cells. (**B**) Quantification of the percentage of oxDJ-1-Cys^106^-SO_3_-positive cells. (**C**) Representative density plot depicting the percentage of iAβ. (**D**) Quantification of the percentage of iAβ-positive cells. Figures represent one of three independent experiments (*n* = 3). The results are reported as the mean ± standard deviation across the trio of independent experiments. Statistical analysis involved a one-way ANOVA followed by Tukey’s test. Statistically significant variations are indicated by * *p* < 0.05, ** *p* < 0.01, and *** *p* < 0.001, and ns = no significance.

**Figure 11:**
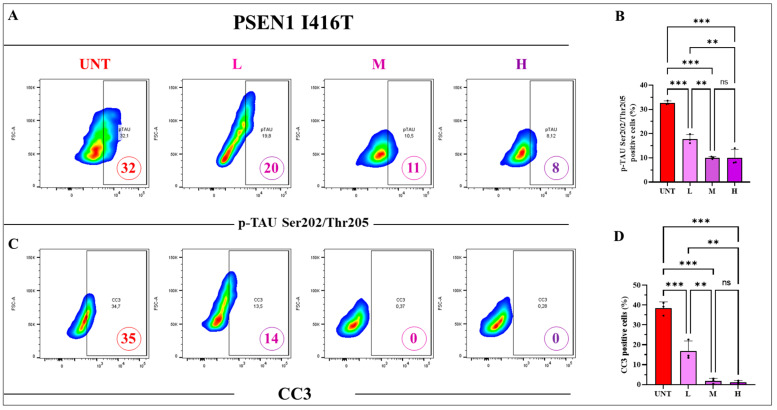
PSEN 1 I416T ChLNs treated with the combination of TM, CU, and SP at low (5, 1, 0.1), middle (10, 2, 0.2), and high (50, 10, 1) concentrations (μM) significantly reduce the phosphorylation of TAU and the activation of the apoptotic marker cleaved caspase 3 (CC3). PSEN1 I416T MenSCs were transdifferentiated and subjected to combined treatments of SP, TM, and CU for 4 days at low (L), middle (M), and high (H) concentrations following the protocols outlined in the Materials and Methods section. (**A**) Representative density plot illustrating the percentage of p-TAU Ser^202^/Thr^205^ as determined by flow cytometry. (**B**) Quantification of the percentage of p-TAU Ser^202^/Thr^205^ -positive cells. (**C**) Representative density plot depicts the percentage of CC3-positive cells. (**D**) Quantification of the percentage of CC3-positive cells. Figures represent one of three independent experiments (*n* = 3). The results are reported as the mean ± standard deviation of 3 independent experiments. Statistical analysis involved a one-way ANOVA followed by Tukey’s test. Statistically significant variations are indicated by ** *p* < 0.01, *** *p* < 0.001, and ns = no significance.

The above results compelled us to evaluate whether a combination of the three reagents might ameliorate the Alzheimer’s phenotype. As shown in Figure 10 and Figure 11, the combination at low (5, 1, 0.1), middle (10, 2, 0.2), and high (50, 10, 1) concentrations was effective in reducing DJ-1-Cys^106^-SO_3_, p-TAU Ser^202^/Thr^205^, and CC3, but only combination H was effective in reducing iAβ. In fact, combination H diminished oxDJ-1 Cys^106^-SO_3_ (Figure 10A,B), iAβ (Figure 10C,D), p-TAU Ser^202^/Thr^205^ (Figure 11A,B), and CC3 (Figure 11C,D) by −86%, −50%, −75%, and −100%, respectively. Except for iAβ, the effect of combination M was comparable to that of combination H (Figure 10 and Figure 11). Given that combination H shows the best positive effect in reducing the four tested markers (Figure 10B,D and Figure 11B,D), this combination was selected as the optimal combination with maximal effects for further experiments.

### 2.9. Combination of Tramiprosate (TM), Curcumin (CU), SP600125 (SP) at High (H) Concentration Together with Anti-Aβ_42_ 1E8 Recovers Dysfunctional ACh-Induced Ca^2+^ Influx in PSEN 1 I416T ChLNs

**Figure 12:**
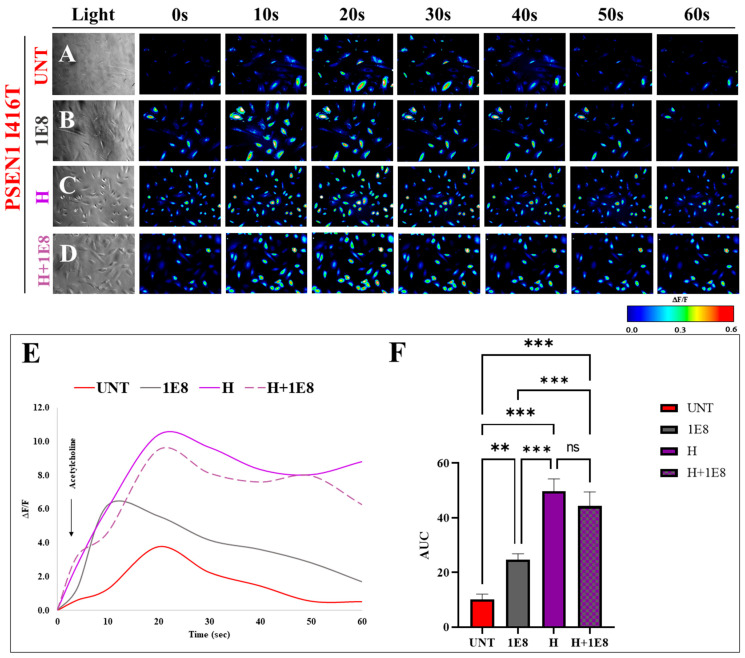
PSEN 1 I416T ChLNs treated with the combination of TM, CU, and SP at high (50, 10, 1) concentration (μM) or together with monoclonal anti-Aβ 1E8 reverse the dysfunctional ACh-induced Ca^2+^ influx. After 7 days of transdifferentiation, PSEN1 I416T ChLNs cultured in regular culture medium for 4 days were left untreated or treated with monoclonal anti-Aβ_42_ 1E8 only, combined TM (50 μM), SP (1 μM), and CU (10 μM), or a mixture of combined TM, SP, and CU together with 1E8 as indicated in the figure. ACh was puffed into the culture at 0s (arrow). Then, the Ca^2+^ fluorescence of the cells was monitored at the specified times. Color contrast indicates fluorescence intensity: dark blue < light blue < green < **yellow** < red. Representative time-lapse images (0, 10, 20, 30, 40, 60 s) of Ca^2+^ fluorescence in response to ACh in PSEN1 I416T ChLNs after 4 days of (**A**) untreated conditions or treatment with (**B**) monoclonal anti-Aβ 1E8, (**C**) combined TM, SP, CU, (**D**) monoclonal anti-Aβ 1E8 and combined TM, SP, CU. (**E**) Graph showing ΔF/F and (**F**) area under the curve (AUC) of cells with and without treatments. The figures represent 1 out of 3 independent experiments. Data are expressed as the mean ± SD; ** *p* < 0.01, *** *p* < 0.001, ns = no significance. Image magnification, 200×.

Finally, we investigated whether a mixture of compounds at H concentration was able to ameliorate the abnormal response of PSEN1 I416T to ACh stimuli. For comparative purposes, monoclonal anti-Aβ_42_ 1E8 was included. When PSEN1 I416T ChLNs were exposed to ACh, cells were irresponsive to that stimulus (average fluorescence change (ΔF/F) = 3.78 ± 0.54 (Figure 12A,E,F) and a mean duration of 20 s each (*n* = 20 ChLN cells imaged, N = 3 dishes)). Upon exposure with ACh and anti-Aβ_42_ 1E8, PSEN 1 I416T ChLNs moderately increased the transient Ca^2+^ influx response to ACh (average fluorescence change (ΔF/F) = 5.59 ± 0.16 and a mean duration of 20 s each (*n* = 20 ChLN cells imaged, N = 3 dishes)) (Figure 12B,E,F). When cells were exposed to combined TM, SP, and CU (Figure 12C), mutant cells significantly increased the response to ACh-induced Ca^2+^ influx with an average fluorescence change (ΔF/F) = 10.42 ± 0.16 and a mean duration of 20 s each (Figure 12E,F). Interestingly, combined TM, SP, and CU together with anti-Aβ_42_ 1E8 (Figure 12D) induced a similar response to ACh-induced Ca^2+^ influx (ΔF/F= 9.52 ± 1.97, mean duration of 20 s each, Figure 12E,F).

Table 2 shows the major findings on the effect of TM, SP, and CU alone or in combination on PSEN 1 I416T ChLNs. 

## 3. Discussion

**Figure 13:**
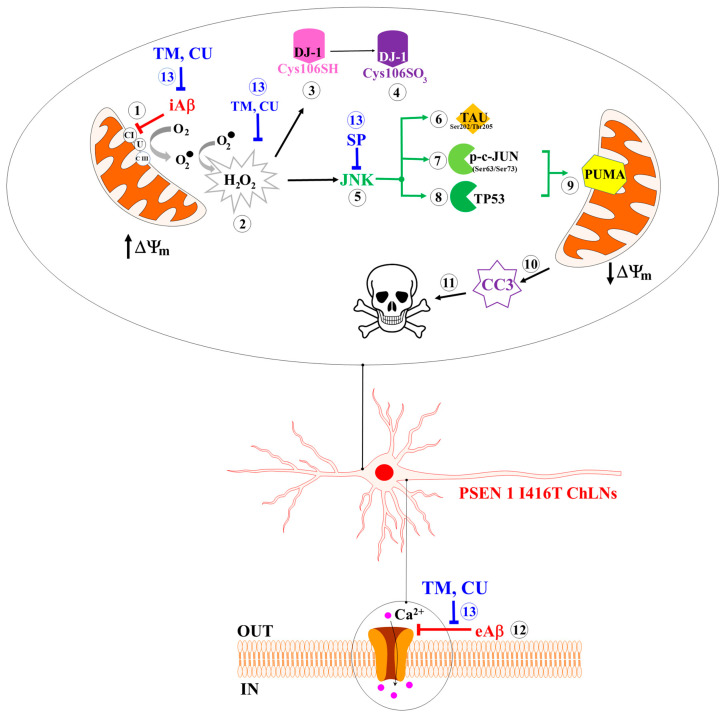
Schematic representation of the mechanism by which combined tramiprosate (TM), curcumin (CU), and SP600125 (SP) reverse the neuropathological Alzheimer’s phenotype in presenilin 1 I416T (PSEN 1 I416T) cholinergic-like neurons (ChLNs).

Previously, it has been shown that ChLNs bearing the mutation I416T in PSEN1 reproduce the typical neuropathology of AD, characterized by the accumulation of iAβ and the phosphorylation of TAU at Ser^202^/Thr^205^ conjointly with the generation of H_2_O_2_, oxidation of DJ-1, phosphorylation of c-JUN at Ser^63^/Ser^73^, loss of ΔΨm, overexpression of TP53 and cleaved caspase 3 (CC3), and failure to respond to ACh-induced Ca^2+^ influx (Figure 13, [33]). In the present investigation, we show that TM, CU, and SP were able to revert those neuropathologic features by inhibiting selectively some molecular targets involved in PSEN1 I416T ChLNs’ demise. Indeed, each agent provided a specific mechanism of action against different targets in mutant cells. However, their combination appeared more effective in reducing all pathological markers. We found that TM significantly increased the viability of cells and reduced the accumulation of iAβ (by −75%) in PSEN1 I416T ChLNs, most probably due to its binding to amino acid side-chain residues Lys^16^, Lys^28^, and Asp^23^ [43] or Arg^5^ Glu^11^ Val^12^ His^13^ Gln^15^ Lys^16^ Leu^17^ Val^18^ Phe^19^ with a moderate binding affinity (−3.3 kcal/mol when compared to EGCG, −5.7 kcal/mol, this work) at the N-terminus of Aβ_42_, leading to the prevention of oligomer seed formation and thus aggregation. This manifest anti-Aβ effect results in a significant decrease in OS and cell death markers. Indeed, we found that TM not only dramatically reduced the generation of ROS (H_2_O_2_, detected as DCF-positive cells), DJ-1 Cys^106^-SO_3,_ and p-c-JUN Ser^63^/Ser^73^, but also increased the ΔΨm and drastically diminished the expression of TP53 and active CC3 in mutant ChLNs. We also tested whether TM was able to act as an antioxidant molecule. Our data clearly showed that TM was devoid of antioxidant capacity. Indeed, TM was unable to participate in the hydrogen atom transfer (HAT) reaction (ORAC assay) or in the single electron transfer (SET) reaction (FRAP assay). These observations support the view that TM works as an anti-amyloid agent rather than an antioxidant molecule. Therefore, TM did not impede p-TAU Ser^202^/Thr^205^. One possible explanation for this observation is that residual iAβ was still capable of inducing phosphorylation of TAU through alternative pathways (e.g., LRRK2 kinase). Interestingly, CU also increased the viability of cells to almost 100% and reduced the proteinopathy (e.g., iAβ, p-TAU Ser^202^/Thr^205^), OS (DCF+ cells, DJ-1 Cys^106^-SO_3_, p-c-JUN Ser^63^/Ser^73^), and cell death markers (e.g., CC3, low ΔΨ_m_) in I416T ChLNs. Effectively, CU inhibited the aggregation of iAβ and reduced ROS, thereby inhibiting the activation of downstream molecular targets such as DJ-1 Cys^106^-SH, c-JUN, and CASP3. Therefore, our observations comply with the notion that CU operates as a bifunctional molecule. On the one hand, it works as anti-amyloidogenic agent [51], probably due to its capacity to inhibit the aggregation of monomeric Aβ peptides (by binding to the aa chain residue Asp^1^ Ala^2^ Arg^5^ Glu^11^ Gln^15^ Lys^16^ Leu^17^ Val^18^ Phe^19^ Glu^22^ Asp^23^, this work) into oligomers or fibrils by disrupting β-sheets within the residue domain _29_GAIIG_33_ and _16_KLVFF_20_ [68], and on the other hand, it operates as antioxidant molecule due to its chemical structure of a non-flavonoid type of polyphenol [69]. Effectively, according to FRAP and ORAC assays, CU was able to engage HAT as well as SET reactions (this work, [70]). It is worth noting that CU showed a higher antioxidant activity than TM. This might reflect a natural property of polyphenols to chemically behave simultaneously as antioxidants and pro-oxidants [71]. Therefore, CU at the non-toxic concentration (e.g., 10 μM) used in our experimental design might operate as a partial antioxidant. Moreover, due to its hydrophobic nature and its very weak water solubility, 10 μM CU was required to obtain its maximum protecting effects. Therefore, improving its solubility in water would require a much lower concentration to attain similar percentages of neuroprotection. Taken together, these results suggest that the phyto-derivatives TM and CU work efficiently by relieving PSEN1 I416T ChLNs from the toxic effect of iAβ, thereby reducing the generation of H_2_O_2_—a molecule that triggers cell death signaling and mitochondrial damage and inactivates CASP3. 

Given that JNK kinase plays an important role in the mechanism of apoptosis [72], its pharmacological inhibition might protect PSEN1 I416T ChLNs from endogenous cellular noxious effects. We report for the first time that JNK inhibitor SP600125 (SP) significantly inhibited p-TAU Ser^202^/Thr^205^, p-c-JUN Ser^63^/Ser^73^, and TP53, which are the three major targets of JNK kinase [73,74,75], and reduced downstream CC3, a critical marker of active CASP3 in dying cells [76]. Interestingly, SP reduced the generation of ROS/H_2_O_2_. This observation suggests that SP might work as an indirect antioxidant, probably by inducing the activation of the nuclear factor erythroid 2-related factor 2 [77,78], a mediator of the cellular antioxidant defense system [79]. Overall, SP showed neuroprotective properties in the mutant ChLNs. 

Until now, no disease-modifying agent has been fully satisfactory against AD/FAD [59] despite several attempts using synthetic or natural products [80]. Here, we demonstrate that combined TM, CU, and SP at different concentrations (i.e., H, M, L) reduced proteinopathy, OS, and cell death, albeit with different strengths, in PSEN 1 I416T ChLNs. Indeed, the combination of three agents at high concentrations (TM 50 μM, CU 10 μM, SP 1 μM) significantly reduced the accumulation of iAβ (e.g., −50%), p-TAU Ser^202^/Thr^205^ (−75%), DJ-1Cys^106^-SO_3_ (−86%), and CC3 (−100%) in PSEN 1 I416T ChLNs compared to untreated mutant cells. Interestingly, except for iAβ accumulation, medium (TM 10 μM, CU 2 μM, SP 0.2) and low (TM 5 μM, CU 1 μM, SP 0.1 μM) concentrations of the combined molecules were also effective in reducing OS and cell death (range from −62% to −100%) markers. These observations suggest that the generation of ROS, OS, and CASP3 are critical factors in reducing the survival of ChLNs, while the accumulation of iAβ is necessary for the death signaling of ChLNs bearing the mutation PSEN 1 I416T. Overall, our data support the view that inhibition of accumulation of iAβ might be the earliest therapeutic strategy to stop iAβ-induced OS and cell death. Moreover, the treatment with combined reagents (TM, CU, SP) might be useful not only for primary prevention strategy against FAD but also in secondary prevention treatments. Although there are now five approved monotherapy alternatives for AD, involving a monoclonal antibody specific for eAβ (aducanumab, lecanemab), memantine, rivastigmine, galantamine, and donepezil, so far, the combination of memantine and donepezil has only been approved for symptomatic treatment in patient with moderate-to-severe AD [81]. While memantine appears to antagonize N-methyl-D-aspartate receptors (NMDARs), a glutamate receptor subfamily, by blocking the receptor-associated ion channel [82,83], donepezil binds and reversibly inhibits acetylcholinesterase (AChE), thus inhibiting hydrolysis of acetylcholine (ACh, [84]). Despite their use individually or in combination [85], none of the available treatments deals with the management of the intracellular Aβ-induced cascade of multi-molecular events as occur in PSEN1 I416T ChLNs. Therefore, combined TM, CU, and SP offer several advantages over other combined approaches, which are basically donepezil-based combination therapy [86]. First, the mechanism of action of each compound—two phyto-derivatives and a pharmacological kinase inhibitor—has a defined inhibitory action such as anti-Aβ accumulation (e.g., TM is equally effective as (=) CU but both agents show highest effect than (>>) SP), anti-p-TAU Ser^202^/Thr^205^ (CU = SP >> TM), anti-DJ-1Cys^106^-SO_3_ (TM = CU >> SP), anti-p-c-JUN Ser^63^/Ser^73^ (SP > TM = CU), and antiapoptotic (SP > TM > CU) activity on the iAβ-induced OS and cell death in ChLNs. Second, combined TM, CU, and SP at their highest concentration tested significantly reduced the typical endogenous accumulation of Aβ, p-TAU, DJ-1, and CC3. Third, the effective intracellular therapeutic effect of combined TM, CU, and SP could be further increased if applied with immunotherapy, which essentially targets eAβ_42_ [32,87], thereby conserving the structure and functionality of ChLNs. Fourth, clinical trials have already proven that TM [45] and CU [88] are tolerable and safe. Interestingly, the addition of valine to TM results in a prodrug named ALZ 801 (valiltramiprosate), which improves pharmacokinetic properties and gastrointestinal tolerability [89]. Nonetheless, ALZ 801 is converted back to TM in vivo, thereby providing high brain levels (40%) that inhibit Aβ oligomer formation in the human brain [44]. On the other hand, LipiSperse^®^ (Pharmako, Frenchs Forest, Australia), a novel dispersion technology system, has increased bioavailability for the supply of CU when compared to a standard CU extract and is safe for humans [90]**.** Finally, combined TM, CU, and SP could be used as an adaptive combination treatment that may prove helpful in various stages of FAD.

Mounting evidence has shown that eAβ_42_ binds to α7 nicotinic acetylcholine receptors (α7nAChRs) with high affinity, affecting calcium homeostasis and cholinergic signaling [91,92]**,** two important parameters involved in AD [93]. This finding might explain why PSEN 1 I416T ChLNs were irresponsive to ACh-induced Ca^2+^ ion influx (this work and [33]). Therefore, a natural strategy to re-establish α7nAChR functionality would be to remove eAβ_42_ or to inhibit the formation of the α7nAChRs/eAβ_42_ complex. Here, we show that TM and CU increase the transient Ca^2+^ influx in PSEN1 I416T ChLNs challenged with ACh. This observation suggests that both agents, through direct binding to monomeric, oligomeric, and/or fibrillar states of eAβ_42_, are able to block the interaction between α7nAChRs and eAβ_42_, thereby restoring the ACh-induced Ca^2+^ influx in mutant cells. Interestingly, a similar result can be achieved with antibodies against eAβ_42_ (e.g., 6E10 [94]), except that TM and CU action might be more meaningful because of their anti-Aβ-accumulation activity not only against eAβ_42_ but also against iAβ (this work, [25]). Remarkably, we found that the combination of TM, CU, and SP at a high concentration was effective in increasing the ACh-induced Ca^2+^ influx in PSEN 1 I416T ChLNs (e.g., 4.86-fold increase in area under the curve, AUC) compared to untreated mutant cells. Although mutant ChLNs moderately recovered upon exposure to anti-Aβ_42_ 1E8 alone (e.g., 2.41-fold increase in AUC) compared to untreated mutant cells, we found a similar maximum Ca^2+^ influx in PSEN 1 I416T ChLNs as a response to ACh when cells were exposed to a combination involving the mixture of TM, CU, and SP alone or together with anti-Aβ_42_ 1E8. We conclude that combined TM, CU, and SP are more effective than monoclonal anti-Aβ_42_ 1E8 in recovering the Ca^2+^-induced influx in PSEN 1 I416T ChLNs. Therefore, combined TM, CU, and SP are a promising disease-modifying therapy against iAβ and eAβ_42_ in FAD. However, it is worth noting that since TM and CU operate on similar targets, especially in inhibiting Aβ aggregation, in future preclinical tests, it would be practical to test whether the combination of TM and SP or CU and SP might accomplish a neuroprotective effect on PSEN 1 I416T ChLNs similar to that of the combination of TM, SP, and CU reported in the present work.

## 4. Materials and Methods

### 4.1. Transdifferentiation of Menstrual Blood-Derived Mesenchymal Stem Cells (MSCs) into Cholinergic-like Neuron (ChLN) Cells

The differentiation protocol was performed according to ref. [95]. Menstrual blood samples were taken from a healthy (Tissue Bank Code, TBC #69308) woman and an asymptomatic FAD patient (TBC #45000). Menstrual specimen donors provided a signed informed consent approved by the ethics committee of the Sede de Investigación Universitaria (SIU), University of Antioquia, Medellín, Colombia (Act 2020-10854). Informed consent was obtained from all subjects involved in the study. Regular culture medium (RCm) containing low-glucose DMEM and 10% FBS was used to seed MSCs on laminin-coated culture plates at a density of 1–1.5 × 10^4^ cells/cm^2^ for a duration of 24 h. Subsequently, the medium was substituted with cholinergic differentiation medium (also known as Ch-N-Run medium, or Ch-N-Rm) that contained DMEM/F-12 Nutrient Mixture (Gibco cat# 10565018, Grand Island, NY, USA), 1% Fetal Bovine Serum (BSA), and a cocktail of nerve growth factors as published elsewhere [96] at 37 °C for 7 days. The cells were given the designation PSEN1 I416T ChLN following the transdifferentiation procedure. For an extra four days post-transdifferentiation, the PSEN1 I426T ChLN cells (obtained after seven days in Ch-N-Rm) were further cultivated in regular culture medium (RCm), as Ch-N-Rm contains a number of components that could potentially impede the interpretation and measurements of the experiment.

### 4.2. Assay Protocol

Tramiprosate (TM, Abcam cat# ab141116, Cambridge, MA, USA), curcumin (CU, Sigma cat# C1386, St. Louis, MO, USA), and SP600125 (SP, Sigma cat# S5567, St. Louis, MO, USA) were evaluated as potential disease-modifying treatments individually or in combination on PSEN1 I416T cholinergic-like neurons (ChLNs). The MTT^®^ viability test (Aldrich-Sigma, cat# M5655) was used to determine the experimental optimum concentration, which was then verified using previously published data: TM was solubilized in PBS or CU and SP in DMSO (https://www.selleckchem.com/products/; accessed on 1 March 2024) at 50 μM [61], CU at 10 μM [62], and SP at 1 μM [32]. A healthy sample was either left untreated (high viability%) or treated as a positive control (low viability%) with rotenone (RO, 10 μM). Following this, the mutant ChLN cells were split into two groups: (i) PSEN1 I416T cells that had not been treated and (ii) PSEN1 I416T cells that had been treated with CU, TM, and/or SP at the appropriate optimum concentrations, either alone or in combination, at low (5, 1, 0.1), middle (10, 2, 0.2), or high (50, 10, 1) rates. After 4 additional days in RCm, cells were used for different analyses.

### 4.3. MTT (Thiazolyl Blue Tetrazolium Bromide) Cell Viability Assay

The MTT assay was carried out in accordance with the supplier’s guidelines (Sigma-Aldrich, cat#M5655, St. Louis, MO, USA). Briefly, the culture medium was cautiously removed from each plated well following each treatment. Each well was then filled with 50 µL of serum-free medium and 50 µL of MTT^®^ solution, and the mixture was incubated for three hours at 37 °C. Each well received 150 µL of MTT solvent after incubation. For fifteen minutes, the foil-wrapped plate was shaken on an orbital shaker. Sometimes, the liquid needed to be pipetted in order to completely dissolve the MTT formazan. Within an hour, plate absorbance at OD = 590 nm was measured. In three separate experiments, the evaluation was conducted again. The percentage of cell viability was calculated using the following equation: % Viability = Mean OD(_untreated sample_) − Mean OD_(treated sample)_/Mean OD(_untreated sample_) × 100. Samples were reported in comparison to the negative control (untreated wild-type ChLN cells) considered to have 100% viability.

### 4.4. Immunofluorescence Analysis

The analysis of markers related to AD, oxidative stress, and cell death was performed exactly as described in ref. [32]. Briefly, cells subjected to varying treatments were fixed for 20 min with 4% paraformaldehyde, permeabilized with 0.1% Triton X-100, and blocked with 10% BSA. The cells were incubated overnight with primary antibodies described in Table 3. After thorough rinsing, the cells were incubated with secondary fluorescent antibodies, and the nuclei were stained with 1 μM Hoechst 33342 (Life Technologies, Cat# H3570, Carlsbad, CA, USA). Images were acquired on a Zeiss Axio Vert.A1 equipped with a Zeiss AxioCam Cm1.

### 4.5. Flow Cytometry Analysis

Trypsin was used to separate the cells after each treatment, and the cells were centrifuged for ten minutes at 2000 rpm. The cells were then permeabilized for 30 min using 1.5% BSA and 0.2% Triton X-100 after being cleaned with PBS. Next, primary antibodies were used against the N-terminal end of the bA4 polypeptide, phospho-TAU, oxidized DJ-1, p-c-Jun, and cleaved caspase 3 (Table 3) at 1:200. Secondary fluorescent antibodies were diluted 1:500 and incubated with the cells after a thorough rinse. Utilizing a BD LSRFortessa II flow cytometer (BD Biosciences), fluorescence analysis was carried out. Cells without primary antibodies served as a negative control. Ten thousand events were collected for evaluation, and FlowJo 7.6.2 data analysis software (TIBCO^®^ Data Science) was used to produce quantitative data and visualizations. The cell population (found using forward scatter analysis, Y-axis) that exceeded the negative control’s baseline fluorescence (488 nm or 594 nm, X-axis) was used to perform the event analysis. As a result, event analysis was used to construct density plots, where cells in the quadrant indicated the cell population that fluoresced above the baseline.

### 4.6. Evaluation of Intracellular Hydrogen Peroxide (H_2_O_2_) by Fluorescence Microscopy

We utilized 2′,7′-dichlorofluorescein diacetate (5 μM, DCFH_2_-DA; Invitrogen, Carlsbad, CA, USA) as described in ref. [32] to measure the intracellular H2O2 levels. ChLNs were kept for four days in RCm. Following this, cells (5 × 10^3^) were treated for 30 min at 37 °C in the dark with the DCFH_2_-DA reagent. After the cells were cleaned, fluorescence microscopy image analysis was used to measure the DCF fluorescence intensity [97]. In three separate experiments, the evaluation was conducted again. Hoechst 33342 staining compound at 0.5 μM was used to stain the nuclei.

### 4.7. Analysis of Mitochondrial Membrane Potential (ΔΨ_m_) by Fluorescence Microscopy

The ΔΨ_m_ in ChLNs were analyzed in accordance with ref. [32]. For four days, ChLNs were cultivated in normal culture medium (RCm). Following this, 5 × 10^3^ cells were cultured with the bright red MitoTracker compound (cat# M22426, Invitrogen, Waltham, MA, USA), which passively fuses and accumulates in active mitochondria (20 nM, final concentration) for 20 min at room temperature in the dark. After that, PBS was used twice to wash the cells. Using fluorescence microscopy image analysis, MitoTracker’s fluorescence intensity was ascertained [97]. In three separate experiments, the evaluation was conducted again. Hoechst 33342 staining compound at 0.5 μM was used to stain the nuclei.

### 4.8. Intracellular Calcium Imaging

With a few modest adjustments, changes in the intracellular calcium (Ca^2+^) concentration brought on by cholinergic stimulation were assessed in accordance with the references [98,99]. The measurement was performed using the fluorescent dye Fluo-3 (Fluo-3 AM; Thermo Fisher Scientific, cat: F1242, Waltham, MA, USA). DMSO (1 mM) was used to dissolve the dye and create a stock solution. The stock solution was diluted in neuronal buffer solution [98,99] prior to the tests. The dye’s working concentration was 2 μM. PSEN1 I416T ChLN cells were treated with the dye-containing NBS for 30 min at 37 °C. This was followed by five washings. On the fourth day post-transdifferentiation, acetylcholine (ACh, final concentration 1 mM) induced intracellular Ca^2+^ transients. Measurements were taken with the microscope’s 20× objective. The visual field of the camera was divided into multiple areas of interest (ROIs). The fluorescence intensity measured in one of the ROIs—which was cell-free—was regarded as background fluorescence (F_background_). Pseudocolorescent was used to depict the fluorescence intensities (and consequently, Ca^2+^ levels) in relation to the recorded time dependency of fluorescence emission. The resting fluorescence intensities (F_rest_) from ROIs containing cells were calculated as the average of points recorded during a consecutive 10 s period prior to acetylcholine addition. The value of F_bg_ was obtained from the cell-free ROI in order to calculate changes in average fluorescence intensities related to Ca^2+^. Peaks of fluorescence transients were found by averaging six consecutive points and identifying those points that yielded the highest average value (F_max_). The amplitudes of Ca^2+^-related fluorescence transients were expressed relative to resting fluorescence (ΔF/F) and calculated using the following formula: ΔF/F = (F_max_ − F_rest_)/(F_rest_ − F_bg_). Fluorescence intensity was calculated using ImageJ. The term “fluorescence intensity” was used as an indirect indicator of intracellular Ca^2+^ concentration. The evaluation was repeated three times in independent, experimenter-blind experiments.

### 4.9. Photomicrography and Image Analysis

A Zeiss Axio Vert.A1 microscope with an AxioCam Cm1 was used to capture optical microscopy images, and a Zeiss Axio Vert.A1 fluorescence microscope with a Zeiss AxioCam Cm1 was used to capture fluorescence microscopy images. ImageJ software (http://imagej.nih.gov/ij/; accessed on 1 March 2024) was used to analyze fluorescence images. The background was removed, and the figures were converted to 8-bit pictures. Regions of interest (ROIs) were drawn around the nucleus (for transcription factors and apoptosis effectors) or around the cells in general (for cytoplasmic probes), and subsequently, fluorescence intensity was determined by applying the same threshold to cells under control and treatment conditions. Mean fluorescence intensity (MFI) was obtained by normalizing the total fluorescence to the number of nuclei (250 nuclei).

### 4.10. Oxygen Radical Absorbance Capacity (ORAC) Assay

As described in ref. [100], the hydrophilic oxygen radical absorbance capacity (ORAC) assay was carried out. Basically, AAPH (2,2′-azobis(2-amidinopropane) dihydrochloride), a conventional molecule called Trolox, a fluorescent probe called fluorescein, and a peroxyl radical generator were used. Trolox equivalents (TE) per gram of solution (µmol) are the reported ORAC values.

### 4.11. Ferric Reducing Antioxidant Power (FRAP) Assay

The FRAP assay was carried out according to the instructions in ref. [100]. Briefly, the samples were combined with the working FRAP solution at a 1:25 ratio and incubated for 10 min at 37 °C in the dark. The Trolox equivalents (TE)/g of solution (μmol TE/g) represent the FRAP values.

### 4.12. Molecular Docking Analysis

For molecular docking experiments, we employed the protein structure of monomeric Aβ (protein data bank code: 6SZF), which was obtained using X-ray diffraction crystallography. CB-Dock version 2 [63], a cavity detection-guided protein–ligand blind docking online server that makes use of Autodock Vina (version 1.1.2, Scripps Research Institute, La Jolla, USA), was used to carry out the blind molecular docking. The SDF structure files of the tested compounds (tramiprosate, compound CID: 1646; curcumin, compound CID: 969516) were downloaded from PubChem (https://pubchem.ncbi.nlm.nih.gov/; accessed on 1 March 2024). The molecular blind docking was performed by uploading the 3D structure PDB file of Aβ into the server with the SDF file of each compound. We chose the docking positions in the binding pocket that had the highest Vina score for study. The produced PDB files for the molecular docking of every compound were compared to the experimentally verified X-ray structures of the interaction between epigallocatechin 3-gallate (compound CID: 65064) and Aβ [65]. These visualizations were made using the CB-Dock2 interphase or BIOVIA Discovery Studio Visualizer (https://discover.3ds.com/discovery-studio-visualizer-download; accessed on 1 March 2024).

### 4.13. Data Analysis

Two vials, each containing PSEN1 I416T MenSCs, were frozen and cultured, and the cell suspension was pipetted onto separate wells of a 24-well plate at a standardized cell density of 2.6 × 10^4^ cells/cm^2^. Using simple randomization (sampling without replacement), cells (i.e., the biological and observation unit) [101] were randomly assigned to wells. Then, using a similar technique, the wells (i.e., the experimental units) were randomly assigned to treatments. Experiments were performed on three independent occasions (*n* = 3) blind to the experimenter and/or flow cytometer analyst [101]. The data from the three repetitions, i.e., independent experiments, were averaged, and a representative flow cytometry density or histogram plot from the three independent experiments was selected for illustrative purposes, whereas the bars in quantification figures represent the mean ± SD and the three black dots show data points of each experimental repetition. Statistical significance was determined using analysis of variance (ANOVA) followed by Tukey’s post hoc comparison calculated with GraphPad Prism 9 software. Differences between groups were considered significant when a *p*-value was <0.05 (*), <0.001 (**), or <0.001 (***). All data are presented as mean ± SD.

## 5. Conclusions

Recent work by our group has revealed that ChLNs derived from PSEN 1 I416T MenSCs reproduced the pathological markers characteristic of FAD, involving the secretion of Aβ_42_, intracellular p-TAU Ser^202^/Thr^205^, iAβ accumulation, and neural cell death [33]. Here, we have used a form of combination therapy consisting of the multifunctional molecules TM, CU, and SP, which as single agents displayed one or more than one activity (e.g., anti-amyloidogenic, antioxidant, anti-kinase activity), leading to almost complete remission of neuropathological markers of AD and functional recovery of PSEN 1 I416T ChLNs (Figure 13). During the last 30 years or so, the amyloid cascade hypothesis (ACH) has provided an elegant mechanistic explanation for AD. Taken together, our data suggest that a combination of intracellular- and extracellular-based therapies might be more effective than monotherapies for the treatment of FAD [102,103,104]. 

The use of MenSCs as an in vitro model for investigational therapies in FAD offers several advantages. First, human-blood-derived MenSCs are exempt from safety issues and ethically and/or politically controversial issues that other biological sources (e.g., human embryonic stem cells [105]) present. Second, MenSCs are a natural source of cells demanding no reprogramming procedures as required for induced pluripotent stem cells [106]. Third, the protocol for obtaining blood-derived MenSCs is a fast, economical, and safe (although vulnerable to infection) process that increases the efficiency, reproducibility, and quality of the experimental results. Therefore, MenSCs are a manageable, cost-effective, time-saving biologic source. Fourth, MenSCs are suitable for obtaining mesenchymal lineage-derived osteoblast, adipocyte, and chondroblast cells by a differentiation process as well ectoderm lineage-derived nerve cells (e.g., ChLNs) by transdifferentiation in eleven days [96]. Last but not least, WT PSEN 1 and mutant MenSCs have proven to be neuropathologically equivalent to iPSCs [107], and this might facilitate the interpretations of potential therapeutic approaches to speeding up drug discoveries against FAD. Since APOE is predominantly produced by astrocytes and microglia [108], and ChLNs do not express or secrete APOE, we consider that the APOE gene identity is not critical information or does not interfere with the experimental interpretation of the present work. Despite these results, MenSCs have some disadvantages. Menstrual blood is considered medical waste; therefore, its collection is not well-perceived by the female population. Educational programs are required to sensitize women toward the importance of blood-derived MenSCs in medical research. Although WT and mutant ChLNs express typical cholinergic lineage markers such as ChAT and VAChT and respond to ACh-induced Ca^2+^ influx, the ChLNs apparently show no cholinergic morphology under the present culture conditions. Consequently, it is necessary to analyze transcriptome and electrophysiological signatures (e.g., patch clamp method [109]) of ChLNs in order to thoroughly validate our findings. Moreover, PSEN 1 I416T ChLNs showed a reduced mitochondrial metabolic activity according to the MTT assay (50% viability) or low mitochondrial membrane potential (~40%) according to the MitoTracker^®^ assay. These observations suggest that the PSEN 1 I416T mutation induces a dormancy state in mitochondria or that PSEN 1 I416T ChLNs contain a reduced number of mitochondria or an absolute loss of mitochondria. Our data support the former assumption. Indeed, TM or CU was able to statistically increase both the mitochondrial metabolic activity (represented in a high percentage of survival) and ΔΨm in mutant neurons. However, further experiments are necessary to clarify this issue. Interestingly, PSEN 1 I416T ChLNs treated with SP showed comparable iAβ and oxDJ-1 percentage values to untreated mutant cells according to flow cytometry analysis, but an analysis of the morphology of neurons by fluorescence microscopy showed a diminished cellular volume compared to untreated ChLNs. A possible explanation for these observations is that the inhibition of JNK kinase alters the cytoarchitecture of mutant ChLNs [110]. However, additional tests are required to elucidate this matter. Given that our findings are based on PSEN 1 I416T MenSCs obtained from a single patient, the conclusions drawn in the present investigation require verification through additional studies with larger sample sizes and other PSEN 1 mutations.

Finally, our data on the accumulation of iAβ identified by Anti-Amyloid βA4 (Millipore clone 1E8, cat# MABN639) should be analyzed with caution. Indeed, due to the fact that Anti-Amyloid 1E8 specifically recognizes the first two amino acids (i.e., Asp-Ala) of the Aβ peptide amino terminus, it might recognize not only iAβ but also other non-Aβ_42_ fragments such sAPPα; Aβ40; and cytotoxic C-terminal fragments γ-CTF(50), γ-CTF(57), and γ-CTF(59). Therefore, while Mab βA4N-1E8 recognizes the free N-terminus of the Aβ_42_ with high preference, this antibody might cross-react with other APP-derived fragments [111]. 

Early accumulation of intracellular Aβ (iAβ) inhibits the mitochondrial complex I (NADH: ubiquinone oxidoreductase), preventing the electron transfer via flavin mononucleotide (FMN) to coenzyme Q10 (**1**). As a result, the disruption of the electron transport chain concurrently generates anion superoxide radicals (O_2_.^-^) and non-radical hydrogen peroxide (H_2_O_2_, **2**). The H_2_O_2_ compound oxidizes the stress sensor protein DJ-1Cys^106^-SH (**3**) to DJ-1Cys^106^-SO_3_ (**4**) and leads to domino-like pro-death signaling of molecules such as JNK kinase (**5**), which is involved in the pathogenic phosphorylation of TAU at residue Ser^202^ and Thr^205^ (**6)**, activation of the proapoptotic transcription factors c-JUN (i.e., p-c-JUN at Ser^63^/Ser^73^) (**7**), and stabilization of the proapoptotic transcription factor protein TP53 (**8**). In turn, both TP53 and p-c-JUN Ser^63^/Ser^73^ increase the expression of the BH-3-only protein PUMA (**9**). This protein affects the mitochondrial membrane potential (ΔΨ_m_), which releases proapoptogenic proteins (e.g., cytochrome C) following the activation of caspase-3 into cleaved caspase 3 (CC3, **10**). Once active, CC3 is responsible for the dismantling of the neuronal cells, a process known as regulated cell death, apoptosis (**11**). Moreover, eAβ_42_ interacts with cholinergic receptors (e.g., nicotinic (n)ACh receptors) (**12**), hindering the ligand-gated Ca^2+^ ion flux. Upon exposure to combined tramiprosate (TM), curcumin (CU), and SP600125 (SP) (**13**), ChLNs show a significant reduction in the accumulation of iAβ, p-TAU (Ser^202^/Thr^205^), DJ-1Cys^106^-SO_3_, p-c-JUN (Ser^63^/Ser^73^), TP53, PUMA, and CC3 and an increase ΔΨm and transient increase in Ca^2+^ influx in response to ACh. All these features represent the recovery and functionality of PSEN 1 I416T ChLNs. 

## Figures and Tables

**Table 1:** In silico molecular docking analysis of Aβ (PDB: 6SZF) with tramiprosate, curcumin, or epigallocatechin 3-gallate (EGCG).

Submitted Protein PDB *	Submitted Ligand **	Vina Score ***	Cavity Volume (Å^3^)	Center (x, y, z)	Docking Size (x, y, z)	Contact Residue
**6SZF**	Tramiprosate CID 1646	−3.3	283	−8, −6, −3	17, 17, 17	Chain A: **Arg^5^Glu^11^Val^12^**His^13^**Gln^15^**Lys^16^Leu17Val^18^Phe^19^
	Curcumin CID 969516	−5.6	283	−8, −6, −3	26, 26, 26	Chain A: Asp^1^Ala^2^Arg^5^Glu^11^Gln^15^Lys^16^Leu^17^Val^18^Phe^19^**Glu^22^**Asp^23^
	EGCG CID 65064	−5.7	283	−8, −6, −3	23, 23, 23	Chain A: Arg^5^**Glu^11^**Val^12^His^13^**Gln^15^**Lys^16^Leu^17^Phe^19^Phe^20^

* According to RCSB Protein Data Base (https://www.rcsb.org/ accessed on 1 March 2024). ** According to PubChem database (https://pubchem.ncbi.nlm.nih.gov/ accessed on 1 March 2024). *** According to CB-dock2: an accurate protein–ligand blind docking tool (https://cadd.labshare.cn/cb-dock2/php/index.php accessed on 1 March 2024). Bold represents the amino acid at the N-terminus of Aβ_42_ involved in the conventional hydrogen bonding with TM, CU, and EGCG.

**Table 2:** Effect of tramiprosate (TM), SP600125 (SP), and curcumin (CU) alone or combination on I416T ChLNs.

Treatment/ Marker	UNT %	TM % ±%	SP % ±%	CU % ±%	Low * % ±%	Middle ** % ±%	High *** % ±%
iAβ	36	9 −75	34 0	9 −75	39 0	38 0	18 −50
p-TAU	32	36 0	15 −53	18 −43	20 −38	11 −65	8 −75
ΔΨ_m_ ^(High)^	64	87 +36	65 0	97 +52	Not done	Not done	Not done
DCF^+^	35	2 −94	27 −22	25 −29	Not done	Not done	Not done
oxDJ-1	29	7 −76	29 0	15 −48	12 −58	0 −100	4 −86
p-c-JUN	42	19 −55	14 −67	29 −31	Not done	Not done	Not done
TP53	26	12 −54	18 −31	31 +19	Not done	Not done	Not done
CC3	34	9 −74	3 −91	20 −41	14 −58	0 −100	0 −100
Ca^2+^ (AUC)	10	40 +300	8 0	38 +280	Not done	Not done	50 +400

The combination of different treatments of TM, CU, and SP at low * (5, 1, 0.1), middle ** (10, 2, 0.2), and high *** (50, 10, 1) concentrations (μM); % represents the mean value of three independent experiments (*n* = 3); ±%, +% = percentage increase with respect to percentage of untreated cells, −% = percentage decrease with respect to percentage of untreated cells; grey shadowed boxes represent the most effective combination for reducing the neuropathological phenotype in familial Alzheimer disease (FAD) PSEN1 I416T cholinergic-like neurons (ChLNs).

**Table 3:** List of antibodies used for immunofluorescence (IMF) staining and for flow cytometry (FC) analysis.

Antibody	Dilution IMF (FC)	Company, Cat#, RRIDs
Differentiation Markers		
Goat anti-ChAT	1:200 (1:200)	Millipore, (Burlington, MA, USA) cat# AB144P, AB_2079751
Rabbit anti-VAChT	1:500 (1:200)	Sigma-Aldrich, Cat# SAB4200559, AB_2910560
Protein Aggregation Markers		
* Mouse anti-Amyloid β A4 clone 1E8	1:500 (1:200)	Millipore clone 1E8, cat# MABN639
Rabbit anti-total TAU	1:500 (1:200)	Sigma-Aldrich, cat# T6402
** Mouse anti-phosphorylated TAU	1:500 (1:500)	Thermo Fisher Scientific, cat# MN1020 (AT8)
Oxidative Stress Markers		
*** Rabbit anti-oxidized DJ-1- ox(Cys^106^)DJ-1	1:500 (1:200)	Abcam, cat# ab169520
Proapoptotic Markers		
Mouse anti-P53	1:500 (1:200)	Millipore, cat# MA5-12453, AB_628082
§ Goat anti-phospho-c-Jun	1:500 (1:200)	Santa Cruz, (Dallas, TX, USA) cat# sc-16312, AB_627262
Rabbit anti-caspase-3	1:500 (1:200)	Millipore, cat# AB3623
Secondary Antibodies		
DyLight 488 horse anti-rabbit	1:500 (1:500)	Vector laboratories, (Newark, CA, USA) DI 1088
DyLight 594 horse anti-rabbit	1:500 (1:500)	Vector laboratories, DI 1094
DyLight 488 horse anti-goat	1:500 (1:500)	Vector laboratories, DI 3088
DyLight 594 horse anti-goat	1:500 (1:500)	Vector laboratories DI 3094
DyLight 488 horse anti-Mouse	1:500 (1:500)	Vector laboratories DI 2488
DyLight 594 horse anti-Mouse	1:500 (1:500)	Vector laboratories, DI 2594

* This monoclonal antibody is specific for the first 2 amino acids (i.e., Asp-Ala) of the amyloid beta (Aβ) peptide amino terminus. ** This antibody is specific for phospho-TAU (Ser^202^/Thr^205^). *** This recombinant monoclonal antibody is specific for PARK7/DJ1—oxidized (Cys^106^-SO_3_). § This monoclonal antibody is specific for phospho-c-Jun (Ser^63^/Ser^73^). Research Resource Identifiers, RRIDs.

## Data Availability

All datasets generated for this study are included in the manuscript.
